# Mechanical transfer of honey bee (Hymenoptera: Apidae) virus sequences to wax by worker traffic and aerosolization

**DOI:** 10.1093/jisesa/ieaf037

**Published:** 2025-05-22

**Authors:** Megan J Colwell, Stephen F Pernal, Robert W Currie

**Affiliations:** Department of Entomology, Faculty of Agriculture and Food Sciences, University of Manitoba, Winnipeg, Manitoba, Canada; Department of Entomology, Faculty of Agriculture and Food Sciences, University of Manitoba, Winnipeg, Manitoba, Canada; Beaverlodge Research Farm, Agriculture and Agri-Food Canada, Beaverlodge, Alberta, Canada; Department of Entomology, Faculty of Agriculture and Food Sciences, University of Manitoba, Winnipeg, Manitoba, Canada

**Keywords:** honey bee virus, black queen cell virus, deformed wing virus, Israeli acute paralysis virus

## Abstract

Honey bees (*Apis mellifera* L.) are of undeniable value to agriculture. However, increased mortality of honey bees, mostly due to winter losses associated with parasites and pathogens, have put strain on the apiculture industry. Advancing our knowledge of honey bee viruses and their interactions within the colony environment is vital in mitigating their effect on honey bee health. Our study examined virus sequences detected on beeswax sampled from empty colonies which died during the previous winter. Based on a cage study using virus-containing bees, we confirmed that the introduction of BQCV sequences to wax foundation was possible through workers walking on, and contacting, comb surfaces (worker traffic). Furthermore, we found that BQCV may aerosolize within an incubator to contaminate wax at detectable levels among independent cages. A second cage study explored the potential effects of virus aerosolization on transmission between groups of adult worker bees within cages, having no direct contact. This experiment did not support aerosol transmission between groups of bees in confined spaces. Further work on waxborne virus transmission within colony environments, and potential effects of aerosolization under a wider array of conditions, is crucial to broadening our knowledge of honey bee virus transmission. Our work also highlights potential dangers for beekeepers re-using equipment from dead colonies.

## Graphical Abstract

**Graphical Abstract:**
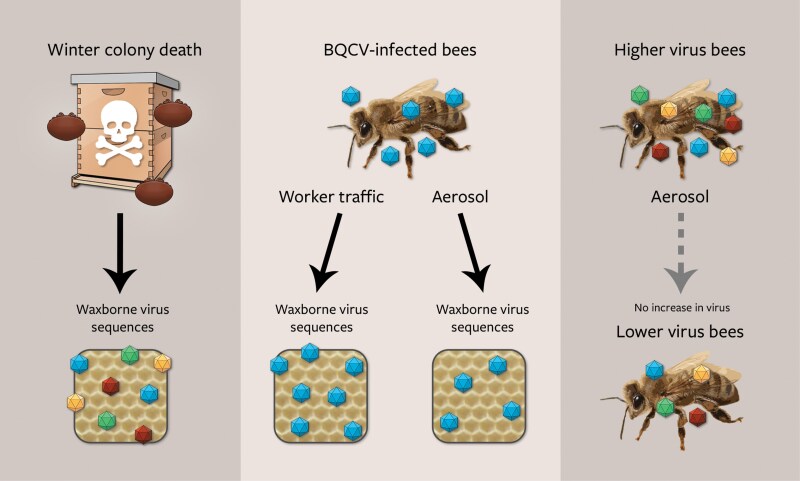

## Introduction

European honey bees (*Apis mellifera* L.; Hymenoptera: Apidae) are an integral part of agricultural pollination worldwide (Delaplane and Mayer 2000, Winfree et al. 2011), as well as providing valuable products such as honey and beeswax. However, an increase in annual losses of honey bee colonies in the last 2 decades has put growing pressure on beekeepers to replace lost stock and meet demand for commercial pollination services (Aizen and Harder 2009, Gallai et al. 2009, vanEngelsdorp and Meixner 2010). In temperate climates, those declines are primarily due to winter losses, specifically, those colonies that die before spring and those that are not large enough to be commercially viable in spring (Currie et al. 2010, vanEngelsdorp et al. 2015, Gray et al. 2020, Ferland et al. 2022).

Research has consistently shown that viruses are linked to honey bee colony losses (vanEngelsdorp and Meixner 2010, Cornman et al. 2012, Carreck et al. 2015). There are in excess of 24 described honey bee viruses, with novel viruses being detected and described with greater frequency than ever before as a result of advances in metagenomic techniques (Remnant et al. 2017, Galbraith et al. 2018, Ray et al. 2020). Honey bee viruses can infect honey bees at any stage in their life cycle and can be transmitted vertically or horizontally (Chen et al. 2006a, 2006b, Yanez et al. 2020). Virus transmission routes in honey bee colonies are incredibly complex, and made even more so by the number of viruses that affect honey bees.

Varroa (*Varroa destructor* Anderson and Trueman) is the most important horizontal vector of several honey bee viruses and also serves as a biological host for some of these pathogens (Chen et al. 2004, Ongus et al. 2004, Shen et al. 2005, Gisder et al. 2009, Francis et al. 2013). Varroa mites and the viruses they vector are recognized as being a major driver of colony losses, if not the major driver (Guzmán-Novoa et al. 2010, Dainat et al. 2012, Carreck et al. 2015, Steinhauer et al. 2018).

Deformed wing virus (DWV) is an economically important honey bee virus and one of the most commonly detected viruses in the United States and Canada (Desai et al. 2015, Fahey et al. 2017). Levels of DWV and its virulence are positively correlated with varroa infestations (Bowen-Walker et al. 1999, Shen et al. 2005, Yang and Cox-Foster 2005, Carreck et al. 2015, Di Prisco et al. 2016, Kang et al. 2016). DWV is comprised of at least 3 major strains in honey bees: A, B, and C (Mordecai et al. 2016a, Bradford et al. 2017). The DWV-B strain, formerly known as *Varroa destructor* virus (VDV-1), is associated with varroa-mediated transmission and widely thought to be more virulent than the other strains, including a correlation with winter losses (Zioni et al. 2011, McMahon et al. 2016, Natsopoulou et al. 2017, Ryabov et al. 2017, Zhao et al. 2019). Contradictory evidence from the United Kingdom shows that DWV-A is more virulent and possibly excludes DWV-B; however, McMenamin and Flenniken (2018) suggest this may be due to selective breeding and resistance in a geographic area rather than a true difference in virulence (Mordecai et al. 2016b).

Black queen cell virus (BQCV) is the other most commonly detected virus in North America (Desai et al. 2015, Fahey et al. 2017). BQCV also infects worker bees, though workers do not show the same symptoms as queen brood do. There is evidence that BQCV can be transmitted vertically and horizontally in honey bees (Chen and Siede 2007). Whereas BQCV has been detected in varroa mites, BQCV is not thought to be vectored by varroa (Locke et al. 2012, Mondet et al. 2014, Yanez et al. 2020). BQCV’s high prevalence in honey bee populations, and lack of connection with varroa, makes it an interesting contrast to DWV for studying virus dynamics.

Israeli acute paralysis virus (IAPV) is another commonly detected virus in both Canada and the United States (Chen et al. 2014, Desai et al. 2015, Fahey et al. 2017). IAPV is linked to colony collapse disorder and winter loss and is also vectored by varroa mites (Cox-Foster et al. 2007, Di Prisco et al. 2011, McMenamin and Genersch 2015, Desai and Currie 2016).

While much attention has been paid to the role of varroa mites in virus transmission, there are other potential transmission routes requiring investigation. Virus transmission via contaminated substrates contacted by bees is poorly understood. There is evidence that honey bee viruses spillover into native and wild bees, suggesting that flowers may act as fomites (Zhang et al. 2012, Alger et al. 2019). These viruses have been found in flower pollen and pollen loads of honey bees (Singh et al. 2010, Chen et al. 2014, Mazzei et al. 2014, Gauthier et al. 2015, Ravoet et al. 2015). This suggests that flowers may act as viral hotspots. Research has made it more apparent that the overall path of honey bee virus transmission is broader than once assumed.

Understanding the role of wax comb in virus transmission is an emerging topic in studying honey bee virus transmission. A common practice in beekeeping is to move comb among colonies, including reusing frames after the death of a colony. Consequently, the potential function of wax in virus transmission both within and among colonies is an important area to investigate. Interestingly, a 2008 study found that a honey bee pest, small hive beetle (Murray, *Aethina tumida*, Coleoptera: Nitidulidae), tested positive for DWV after being exposed to DWV-contaminated wax (Eyer et al. 2008). Gamma irradiation has been tested as a prospective treatment to control viruses on beeswax, whereby newly emerged bees reared in irradiated wax comb had lower levels of DWV compared to those from non-irradiated wax (de Guzman et al. 2017). Nevertheless, a subsequent study showed inconsistent results of comb irradiation, with greater pollen stores and adult bee population size in irradiated colonies in the first year but not the second (de Guzman et al. 2019). A further study focused on DWV-A found that honey bees had increased DWV-A levels after exposure to experimentally contaminated wax foundation at levels not significantly different from bees exposed to contaminated honey or pollen (Schittny et al. 2020). All of the above results suggest that wax itself could be an important route of transmission in honey bee colonies.

Waxborne viruses represent a relatively new area of research, and as such, basic research must be done to create a foundation for future work. We used a method to test wax directly for honey bee virus sequences to examine the mechanisms through which waxborne viruses are deposited on comb. We had 3 objectives: (i) to detect waxborne viruses from colonies having died during winter storage (hereafter, winter loss colonies); (ii) to determine if “worker traffic” could introduce viruses to a beeswax surface; and (iii) to test the possibility of aerosol or contact transmission between highly infected bees to control worker bees.

## Materials and Methods

### Experimental Design and Sampling

#### Winter Loss Wax Study

Wax was sampled from hives that died over the winter of 2017 to 2018. Hives were moved into an indoor wintering facility on 10 November 2017, which was operated according to standard commercial beekeeping practices for the region (Gruszka et al. 1998). A total of 195 hives were wintered in the fall of 2017 and 151 survived until the spring. Forty dead hives were selected and sampled when all colonies were removed from the building, on 16 April 2018. Hives were arbitrarily chosen based on their mite infestation levels from the previous autumn; 20 from the lower half of the range and 20 from the upper half (see Results for infestation levels). Brood frames were inspected and samples collected from areas of wax comb without dead brood or stored food. A 2.0-ml microcentrifuge tube was pressed into and dragged through the comb until the tube was nearly full of loosely packed wax. One tube was collected from one frame from each winter loss hive. Wax samples were then stored in a -80 °C freezer.

#### Worker Traffic Cage Study

**Fig. 1.**
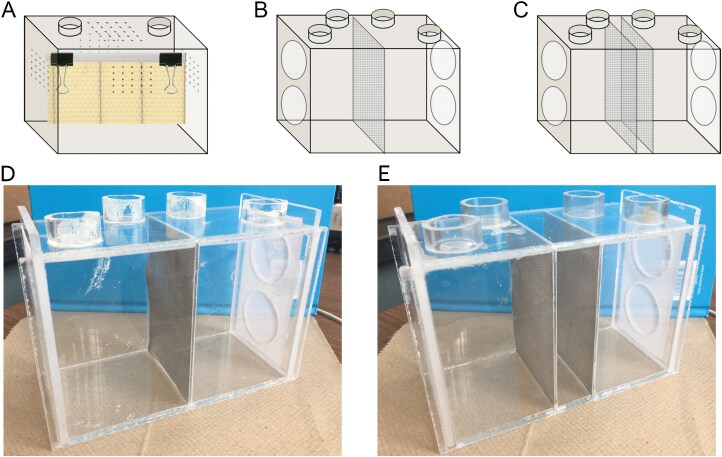
Diagram of cage types for (A) the worker traffic cage study, and (B and D) single mesh and (C and E) double mesh cages for the aerosol cage study. Worker traffic cages show the set-up with wax foundation, all sides and the top have ventilation holes, and the front panel slides up to provide an access door. Aerosol cages show the set up with metal mesh separation walls and ventilation holes covered in fabric mesh. The walls with ventilation holes in the aerosol cages are also doors that slide up, one on each end. All sucrose solution and water ports are shown empty, and the bag surrounding each cage and associated ventilation apparatus is not shown for the aerosol cages. Worker traffic cages were 10.8 cm × 7.7 cm × 15.3 cm, aerosol cages were 10.5 cm × 7.9 cm × 16.8 cm. Images (D and E) showing detail of cages with screens, which allowed aerosol movement but prevented mite transfer between cage sides.

In July 2015, commercially available wired wax foundation sheets (Mann Lake Ltd., Hackensack, MN; 8 ½” deep for standard sized Langstroth hives) were cut to size (14 cm × 7 cm) using a sterilized scalpel and a sterilized wire cutter and assigned to one of 4 treatment groups: (i) caged bees with “high” virus levels (HV), (ii) caged bees with “low” virus levels (LV), or (iii) cages with wax but without bees. A final treatment (iv) was wax foundation held outside cages and the incubator itself, as a control for airborne transmission. LV and HV describe relative virus loads based on mite infestation levels within this experiment, with an assumption that colonies with significantly lower mite levels would also have lower virus levels than colonies with higher mite infestations. Each treatment group included 8 replicates for a total of 24 cages and 8 non-cage controls. Plexiglass cages (10.8 cm × 15.3 cm × 7.7 cm) with a vertical-sliding door were used for the first 3 treatments (Fig. 1A). Cages had 1 mm ventilation holes in groups of 25 drilled through all vertical sides as well as the top, and each cage had 2 ports for syringes to allow feeding on the top side. Wax sheets were hung in cages using a ~1 cm wide binder clip on each end of a 14-cm-long report cover spine bar (Gemex Slide Clamp Spines for Report Covers, Granby, QC), and fine metal wire to suspend the wax sheet near the top of the cage to hold it approximately in the center of the cage. The metal wire loops were drawn through ventilation holes on opposite sides of the cage and twisted to secure the wax sheets. The final treatment group of wax foundation was not placed in cages or an incubator; 8 sheets of wax foundation were stored in a drawer in a separate room from the incubators.

Bees for the HV and LV cage treatments were sourced from 8 colonies each from 2 separate apiaries. HV bees were sampled from a research apiary (49°48'33.0''N, 97°07'35.2''W) managed to maintain high varroa mite infestations, with a mean infestation rate of 18.4% on adult workers sampled in spring. LV bees were sampled from an apiary (49°50'11.7''N, 97°17'49.1''W) treated for varroa 2 mo prior, with a mean infestation rate of 1.4% sampled in the following autumn. The 2 apiaries were ~11 km apart from each other to prevent mixing of the bees. Bees were collected in the morning, allowed to homogenize within treatment, sorted into cages in the early afternoon, and the experiment initiated in late afternoon of the same day, 17 July 2015. Worker bees from each source (HV or LV) were collected by shaking a single brood frame from each of the 8 source colonies in an apiary into a large hoarding cage. Queens were located and, if necessary, moved to another frame prior to shaking. Bees in the hoarding cages were lightly sprayed with a water and vanilla extract solution between shaking a new frame of bees. The 2 hoarding cages were kept in one incubator for approximately 3 h to allow homogenization. The incubator was set at 30 °C, kept at 75% RH using a basin of open water on the bottom of the incubator, and kept dark (Williams et al. 2015). Each cage with wax was randomly assigned to a treatment, and those which had a “bee” treatment received 300 workers introduced to cages after the homogenization period. Bees from each source were anesthetized briefly with CO_2_ and counted into cages in batches while immobilized. Each of the 24 cages was supplied with 20 ml sucrose solution (1:1 w/w distilled water and sucrose) and 15 ml distilled water in 20 ml disposable plastic syringes secured into cage ports with masking tape (BD Luer-Lok, BD, Franklin Lakes, NJ). Cages were then randomly positioned on one of 2 shelves in the incubator. Shelves were covered with aluminum foil and cages were surrounded by a petroleum jelly perimeter to prevent possible movement of varroa among cages. Incubator conditions were as described above for the homogenization period. Temperature, humidity, sucrose, and water consumption, as well as worker bee mortality, were recorded daily with cages removed individually from the incubator for examination. Sucrose and water were replaced *ad libitum*. Dead bees were counted and removed daily to prevent possible contamination of the living bees. The cage door was kept as low as possible to minimize bee escape during this process.

The experiment concluded after a final inspection on the seventh day, on 24 July 2015. A total of 18 bee samples, comprised of 30 surviving bees per cage, and 32 wax sheet samples were collected and stored separately at −80 °C for quantitative analysis of viruses. Strict protocols were in place to minimize possible contamination during sample collection, including sterilizing all instruments with 95% ethanol, spraying work surfaces with 95% ethanol, and replacing nitrile gloves between each sample taken. Wax and bee samples were only tested for BQCV because it was the only optimized primer for detecting viruses extracted from beeswax at the time the experiment was conducted in 2015.

#### Contact vs. Aerosol Cage Study

**Fig. 2.**
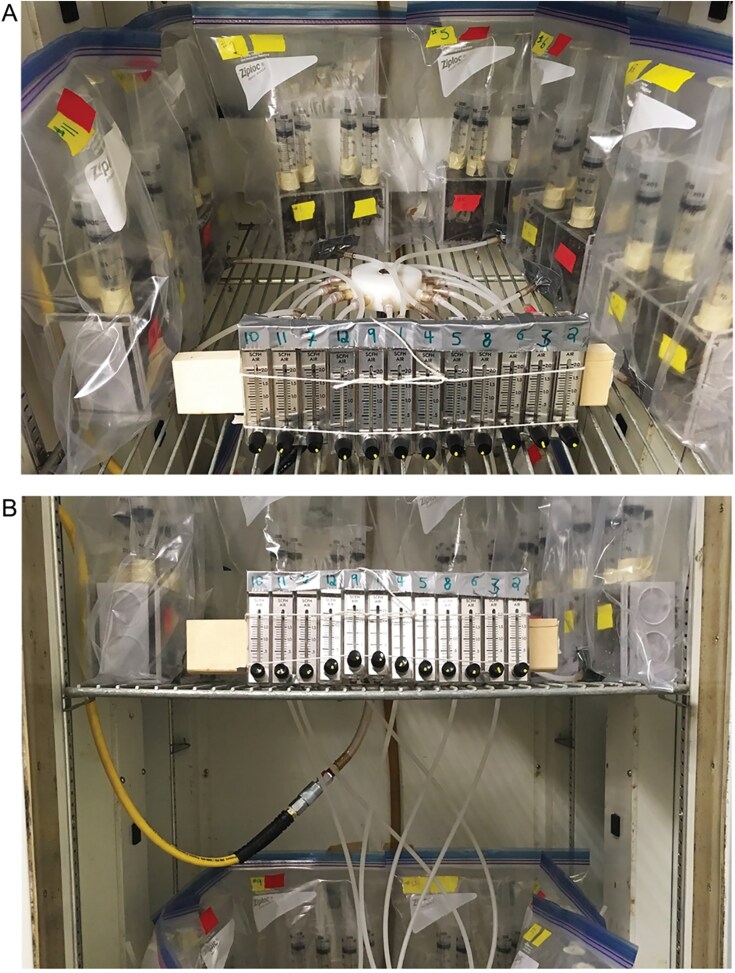
Photographs of the aerosol cage study incubator setup. Showing (A) the hub splitter and individual flowmeters for each cage/bag on the two shelves, and (B) the yellow hose that supplied air from the compressor to the cages via the hub splitter.

Two types of cages were used in the aerosol experiment, each with 2 equal-sized compartments separated by either a single metal mesh wall, or 2 metal mesh walls (Fig. 1B and C; stainless steel woven wire mesh, wire diameter 0.15 mm, mesh aperture 0.28 mm). The single mesh cages were designed to allow contact and trophallaxis between the 2 sides of the cage. The double mesh cages were designed to prevent direct contact or trophallaxis between the 2 sides of the cage with a 2.5-cm gap between the metal mesh (consequently, the bee spaces of double mesh cages were smaller in volume than single mesh cages). Both cage designs allowed complete air exchange between the 2 sides. Additionally, the mesh was fine enough to prevent movement of varroa mites within cages (Fig. 1D and E). These cages were used to create 3 main effect treatment groups: (i) control cages with LV bees on both sides of a double mesh, (ii) aerosol cages with LV bees and HV bees on opposing sides of a double mesh, and (iii) contact cages with LV bees and HV bees on opposing sides of a single mesh. LV and HV describe relative varroa loads from source colonies within this experiment; mite levels were not manipulated in cages. Each treatment group included 8 replicates for a total of 24 cages. Cages were enclosed in sealed plastic bags and provided with individual air supply tubes from an air compressor (Speedaire Portable Electric Air Compressor, 30 gal.) outside of the incubator (Fig. 2A and B). This extra feature meant fewer cages could fit at one time than in the previous cage study, and accordingly the experiment took place over 2 separate weeks with half of the cages (12) each week, with treatments balanced across the 2 wk. Cages were randomly assigned to the 2 shelves of the incubator. Plexiglass cages of similar dimensions as the worker traffic cage study were used. Cages had 2 doors on opposing ends, which slid open vertically. The doors had two 3-cm diameter circles cut out of the plexiglass and covered with fine mesh material to allow ventilation. A piece of fiberglass window screening was cut to size (~6.25 to 7.5 cm × 7 cm, width dependant on cage type) and hot glued to the top center of the cage to provide a substrate for the bees to cluster on.

Bees for the HV and LV sides were collected in the same way to the previous cage study. In this instance, the 2 apiaries were ~2 km apart from each other. HV colonies (49°48'15.8"N, 97°09'17.9"W) had a mean infestation rate of 3.1% ± 2.7 per colony sampled in July, and LV bees were sourced from an apiary (49°48'48.9"N, 97°07'03.8"W) of bees hived from New Zealand packages in May 2017, with a mean infestation rate of 1.3% ± 1.5 per colony sampled in the following autumn. Bees for the LV group were from New Zealand packages hived on 7 May 2017. Bees were collected and allowed to evenly mix within treatment, then introduced into cages. Each side received 150 bees, for a total of 300 bees per cage. Week one started on 23 August 2017 and week 2 on 30 August 2017. Both sides of each cage had syringes for water and sucrose solution. Cages were placed in individual 8 L sealable bags (Ziploc, SC Johnson). Each bag had 2 “ports,” one for air input and one for output. Ports were formed by cutting a hole in duct tape-reinforced squares attached to the bag through which a short length of plastic tubing ~1 cm diameter, ~5 cm length) was inserted and sealed to the bag with additional duct tape. Input ports were located near the bottom and front of the bag, and output ports were at the top and back, relative to the cage. Input ports were attached via tubing to a Dwyer flowmeter (0.5-2.0 SCFH air, Dwyer Instruments, Michigan City, IN) and a splitting hub providing air from an air compressor outside of the incubator (Fig. 2B). Junctions of tubing were sealed with Parafilm M (Sigma-Aldrich). Flow was standardized to 0.057 m^3^/h, which was enough to keep the bags partially inflated. Output ports vented directly into the incubator. Daily checks were done to monitor temperature and sucrose solution and water levels, with twice daily checks of air flow rate. Due to the complex arrangement of the cages in bags, mortality checks were not completed during the experiment. When required, sucrose solution and water was replenished one cage at a time in a well ventilated area nearby.

Both weeks of the experiment concluded after 6 d, on 29 August 2017 and 5 September 2017. A total of 48 surviving workers, pooled from each side of each cage, were collected and stored separately at −80 °C for quantitative analysis of viruses.

### RNA and cDNA

#### Wax Sample Processing

Subsamples of wax from the worker traffic cage study and winter loss wax study were used to perform a wax wash (Colwell et al. 2024), modified from previous studies on viruses in pollen (Aparicio et al. 1999, Singh et al. 2010). RNA was extracted from wax supernatant samples with PureLink RNA Mini Kits (PureLink, Life Technologies, Carlsbad, CA) starting with the RNA purification steps for binding, washing, and elution of RNA. Due to the scarcity of RNA recovered from wax washing, only 100 ng of RNA was used in a volume of 20 µl cDNA. Samples from the winter loss wax study were washed twice in an attempt to obtain more RNA, which proved successful. This RNA was used to synthesize greater quantities of cDNA (100 ng/20 µl), in order to facilitate testing a greater number of more viruses. cDNA synthesis was performed using a kit (iScript cDNA Synthesis Kit, Bio-Rad, Hercules, CA).

#### Honey Bee Sample Processing

Surviving worker bees retained from the conclusion of both cage studies were pooled by cage (or side of cage in the aerosol cage study) and subsamples of 30 bees were processed. Any bees additional to the 30 subsampled were retained, whole, at −80 °C. Bees were crushed with a Geno Grinder (1750 rpm, 3 min with two 9 mm steel beads, Cole-Parmer, Vernon Hills, IL) after cooling with liquid nitrogen and addition of 2.0 ml lysis buffer. RNA was extracted using PureLink RNA Mini Kits according to the protocol for purification from animal tissues. cDNA was synthesized as above, using 1 µg of RNA in a volume of 20 µl cDNA.

### RT-qPCR

Virus quantification was performed using a CFX384 Touch Deep Well Real-Time PCR Detection System (Bio-Rad, Hercules, CA). Assays were done in accordance with the protocol of SsoAdvanced Universal SYBR Green Supermix (Bio-Rad, Hercules, CA).

Due to scarcity, wax samples were run in technical duplicate, and each well had 4 µL of undiluted cDNA containing 20 ng of cDNA synthesized from pooled RNA. Bee samples were run in technical triplicate, and each well had 2 µl of template cDNA containing 50 ng of cDNA synthesized from pooled RNA (1:1 dilution). All plates were run with non-template and no reverse-transcription controls. Samples from the winter loss wax experiment were tested for BQCV, IAPV, DWV (generic), DWV-A, and DWV-B. Samples from the worker traffic experiment were tested for BQCV. Samples from the aerosol cage experiment were tested for BQCV, IAPV, DWV-A, and DWV-B. Honey bee samples were also tested for a housekeeping gene (beta actin).

Standard curves using gBlock gene fragments (Integrated DNA Technologies, Coralville, IA) were run on each virus plate to allow absolute quantification of gene copies. Standard curves were based on concentrations from 10^2^ to 10^7^ BQCV for wax samples (E% = 104 to 112, *R*^2^ = 0.995 to 0.996). Standard curves for honey bee samples with efficiencies of 102% for BQCV (*R*^2^ = 0.999) based on concentrations from 10^4^ to 10^9^, 89% for IAPV (*R*^2^ = 0.998) based on concentrations from 10^3^ to 10^9^, 103% for DWV generic (*R*^2^ = 0.998) based on concentrations from 10^4^ to 10^9^, 96% for DWV-A (*R*^2^ = 0.998) based on concentrations from 10^3^ to 10^9^, 93% for DWV-B (*R*^2^ = 0.998) based on concentrations from 10^4^ to 10^9^. Example standard curves, melt curves, and melt peak figures can be found in Supplementary Figs. S1-S5. Amplifications were performed using a single cycle heated for 3 min at 95 °C, and 40 cycles at 95 °C for 15 s, 55 °C for 30 s, 72 °C for 30 s, and a plate read. Melt curves were recorded at the end of the cycles, increasing from 65 °C to 95 °C by 0.5 °C increments. All primers used in this study can be found in Supplementary Table S1; gBlock sequences can be found in Supplementary Table S2. To verify PCR products, excised amplicons were purified using NucleoSpin Gel and PCR clean-up kit (Macherey Nagel, Duren, Germany). Elutes were sent for Sanger sequencing (Psomagen, Maryland, USA). Obtained sequences were trimmed and compared to nucleotides sequences in the NCBI database by BLAST (Supplementary Table S3).

### Statistical Analysis

Statistical analyses were performed in SAS software version 9.4 (SAS Institute, Cary, NC). We measured virus prevalence (the number of wax or bee samples that tested positive for a variable, in this case virus sequences, divided by the total number of samples tested within an experiment) and mean abundance (the mean number of a pathogen, in this case gene copies of viral sequences per 20 ng of cDNA, divided by the total number of bee hosts or wax sources for each) (Bush et al. 1997). Viral sequence gene copy data were log-transformed after adding 1 as a constant prior to analyses (Pirk et al. 2015). Prevalence of viruses were analyzed using logistic analyses (PROC CATMOD, SAS 9.4) and differences among proportions were compared using maximum likelihood with Bonferroni corrections. A mixed model ANOVA was used to analyze mean abundance data in both the winter loss (colony was treated as the subject and classified as a random variable) and aerosol experiments (cage was treated as the subject and classified as a random variable; PROC MIXED, SAS 9.4). Similarly, a mixed model ANOVA was used to compare treatments in the worker traffic study. Data that did not meet the assumptions related to homogeneity of variance (Levene’s test) were adjusted using Kenward-Rogers degrees of freedom approximation during analysis. Correlations in the worker traffic study were analyzed with Pearson’s product-moment correlation. Statistical differences between the 2 mite levels in the winter loss experiment and BQCV levels in the worker traffic were determined with Student’s *t*-tests. All figures display data based on maximum likelihood values for prevalence and least squares means for abundance, excluding linear regressions showing correlations.

The winter loss wax study was analyzed with PROC MIXED with mite blocking as a main effect, colonies as the subject (random effect), and virus type within colonies as a sub-effect using a compound symmetry covariance structure. The worker traffic study was also analyzed with PROC MIXED as a one-way ANOVA with 4 wax exposure treatments and cage as the experimental unit. The contact vs. aerosol cage study was analyzed with PROC MIXED using cage as the subject and week as a blocking factor; fixed-effects were treatment, cage side within cage, and an interaction of treatment by cage side within cage. Differences among means were compared using Pdiff with Tukey-HSD comparisons, except when examining interactions where a significant interaction was identified using SLICE (SAS 9.4). Viruses were tested separately and each used the most appropriate covariance structure as determined by examining the AIC for several common structures. BQCV was analyzed with autoregressive covariance structure, IAPV with heterogeneous autoregressive covariance structure, and DWV-A with variance components covariance structure.

In the contact vs. aerosol cage study, samples with a Ct value greater than 23.5 for beta actin were excluded from analysis to avoid using samples that had degraded during shipping (Robson-Hyska 2017). There were 9 samples over both weeks with poor beta actin values.

## Results

### Winter Loss Wax Study

**Fig. 3.**
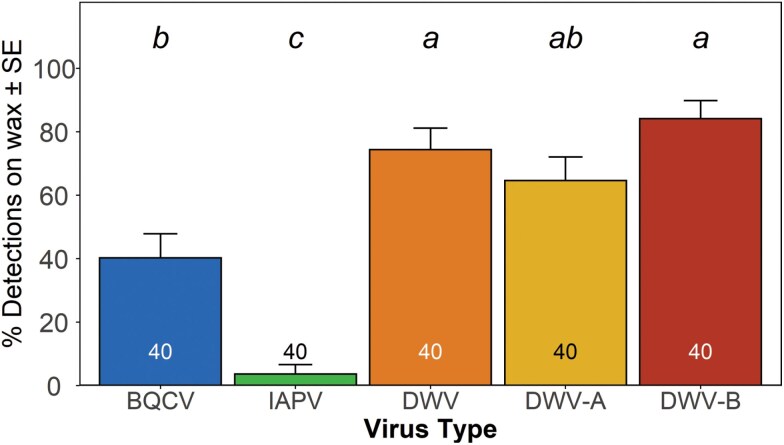
Presence of honey bee viruses detected on winter loss wax comb. Prevalence ± SE of positive detections for BQCV (black queen cell virus), IAPV (Israeli acute paralysis virus), DWV generic (deformed wing virus, generic primer), DWV-A (deformed wing virus, strain A), and DWV-B (deformed wing virus, strain B) per 20 ng of cDNA on winter loss colony wax (all bars *n* = 40). Plotted data are based on maximum likelihood estimates. Standard error was calculated as SE of binomial proportions. Virus types with different letters above bars are significantly different (*P* < 0.05: repeated-measures ANOVA, Bonferroni corrected).

We first tested if wax from winter loss hives had detectable levels of honey bee virus and which viruses were present, determined by detection of viral sequences. Wax from 40 hives that died during indoor wintering were tested for 5 viruses: BQCV, IAPV, DWV (using a generic primer), and the 2 strains DWV-A and DWV-B (using strain-specific primers). There were significant differences in positive detections of these viruses on winter loss colony wax (*χ*^2^ = 35.3, DF = 4, *P* < 0.001). BQCV was present in fewer samples than DWV-B and DWV (generic) but in more samples than IAPV, whereas IAPV was detected in significantly fewer samples than all other virus types (Fig. 3).

Though the difference in mite levels between the 2 groups in our study was statistically different (*t* = 5.6, DF = 38, *P* < 0.001) the range in mite levels was low. The lowest 20 hives had a range of 0.0 to 0.8% varroa infestation (varroa per 100 bees) and a mean of 0.3% ± 0.3, while the highest 20 hives had a range of 0.9 to 6.1% and a mean of 2.3% ± 1.6. Based on the relatively low infestation levels, we subsequently pooled all wax samples for analyses.

**Fig. 4.**
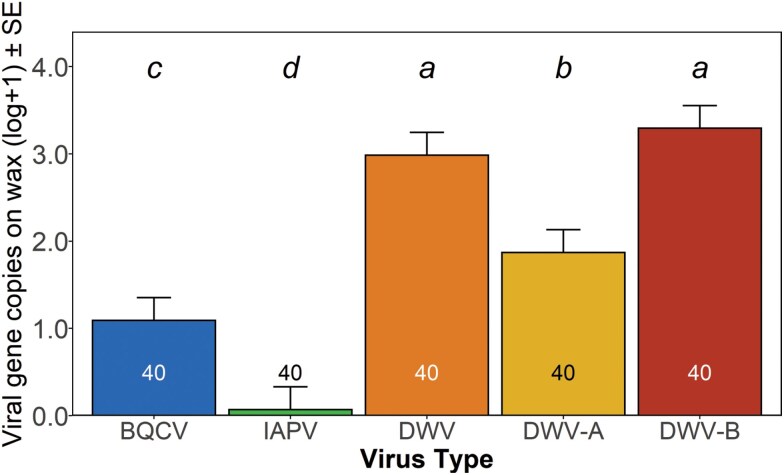
Levels of viruses present on winter loss wax comb. Mean abundance ± SE of BQCV (black queen cell virus), IAPV (Israeli acute paralysis virus), DWV generic (deformed wing virus, generic primer), DWV-A (deformed wing virus, strain A), and DWV-B (deformed wing virus, strain B) gene copies per 20 ng of cDNA on winter loss colony wax (all bars *n* = 40). Displayed data are based on least squares means. Virus types by time with different letters above bars are significantly different (*P* < 0.05: repeated-measures ANOVA, Tukey HSD).

Due to the low variation in mite level in sampled colonies described above, no differences in virus within the mite blocking factor were noted (*F*_1,38_ = 0.5, *P* = 0.5) nor were there any interactions between the mite block and virus (*F*_4,152_ = 0.7, *P* = 0.6). Therefore these were deleted from the model when we compared abundance of the previously mentioned viruses across samples where virus abundance did vary within colonies (*F*_4,152_ = 48.4, *P* < 0.001). DWV (generic primer) and DWV-B were at higher levels on winter loss wax than the other viruses, DWV-A was higher than BQCV and IAPV, while BQCV was present at greater levels than IAPV (Fig. 4).

**Fig. 5.**
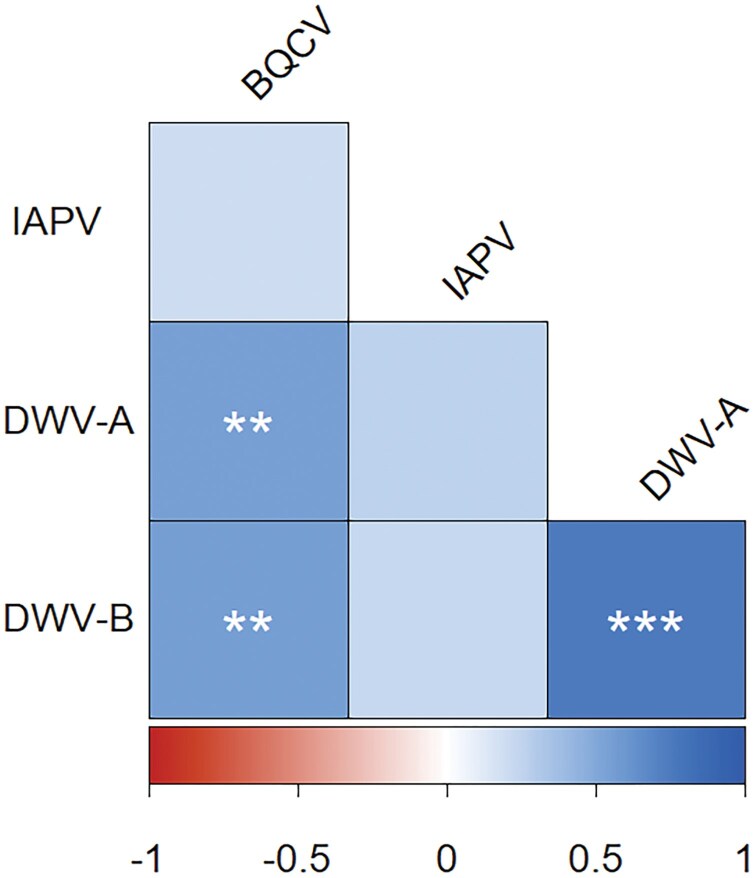
Correlation matrix of virus relationships within winterloss colony wax samples. Asterisks represent a significant correlation (*** = *P* < 0.0001, ** = *P* < 0.001, * = *P* < 0.05: Spearman’s correlation; df = 38). The scale along the bottom shows direction of the correlation (red/left is negative and blue/right is positive), and intensity of color saturation represents the strength of the correlation.

We assumed that if competition among viruses deposited on wax comb was occurring there would be negative correlations among viruses. We tested each virus for possible correlations with the other viruses. Positive correlations among viruses were detected on winterloss colony wax between all viruses except IAPV (Fig. 5).

### Worker Traffic Cage Study

We tested wax foundation in cages to determine if worker traffic was sufficient to introduce a virus to wax at detectable levels and if any honey bee viruses could aerosolize in an incubator. Samples from HV cage bees and wax, LV cage bees and wax, interior incubator control wax, and exterior incubator control were tested for BQCV gene copies (wax *n* = 32 and bee *n* = 16 samples). Prevalence of BQCV was not similar across all sample types (*χ*^2^ = 11.4, DF = 3, *P* = 0.010); with detections in 100% of wax samples from HV cages, LV cages, and interior controls, and 0% on exterior control wax foundation. BQCV was present in all samples of bees, whether HV or LV.

**Fig. 6.**
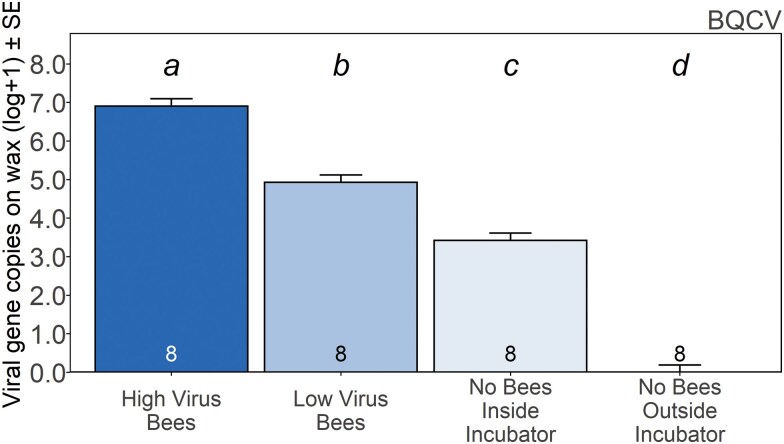
Effect of bee and incubator treatments on virus introduction to wax foundation. Mean abundance ± SE of BQCV (black queen cell virus) gene copies per 20 ng of cDNA on wax from the worker traffic cage experiment (all bars *n* = 8). Plotted data are based on least squares means. Wax sample types with different letters above bars are significantly different (*P* < 0.05: repeated-measures ANOVA, Tukey HSD).

Analyses of BQCV abundance of the 4 wax sample types showed that gene copies significantly differed by treatment (*F*_3,28_ = 237.1, *P* < 0.001). Wax from HV cages had significantly greater virus gene copies of BQCV than wax from LV bees, with both cages containing bees having higher levels than the interior control. There were no positive detections of BQCV on the exterior wax control (Fig. 6). Bees from HV cages had significantly higher mean abundance of BQCV (2.4 × 10^7^ ± 7.1 × 10^6^ gene copies per 20 ng of cDNA) than bees from LV cages (4.7 × 10^5^ ± 3.2 × 10^5^ gene copies per 20 ng of cDNA; *t* = 3.4, DF = 7, *P* = 0.01).

**Fig. 7.**
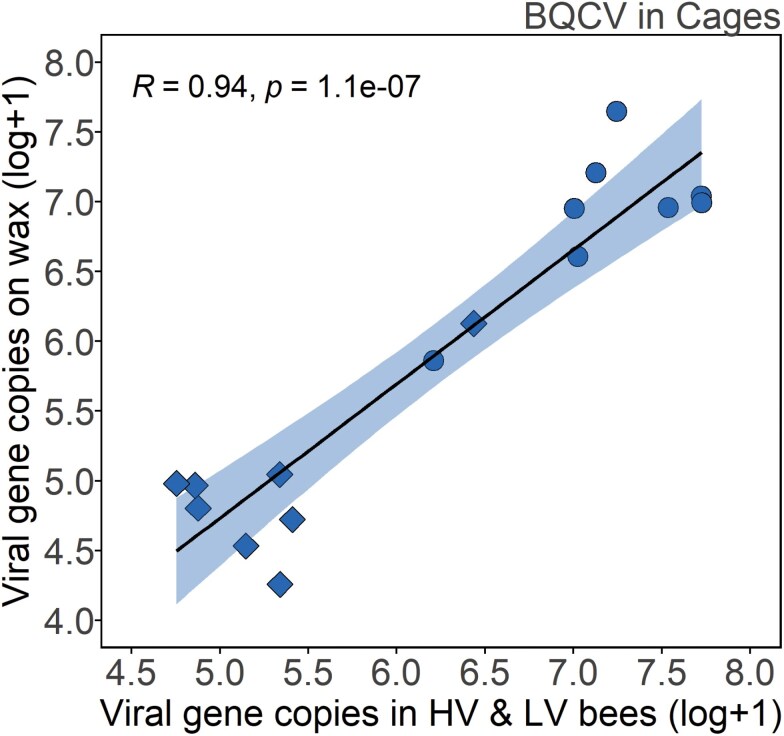
Correlation between BQCV levels on wax and in bees in the worker traffic cage experiment (*n* = 16). There was a positive correlation between BQCV in bees and BQCV detected on wax (*P* < 0.001: Pearson’s correlation). Diamonds represent LV data and circles represent HV data.

There was a significant positive correlation between bees pooled by cage type and waxborne virus levels (*r* = 0.94, *t* = 9.9, DF = 6, *P* < 0.001); Fig. 7).

### Contact vs. Aerosol Cage Study

Finally, we tested bees from HV “donor” and LV “acceptor” sides of cage treatments to determine if aerosol or contact movement of viruses from adult bees on the donor side would result in transmission to adult bees on the acceptor side of cages. Bees from each side of all 24 cages used in the 3 treatments (Aerosol, Contact and Control) were tested for BQCV, IAPV, DWV-A, and DWV-B using absolute viral starting quantity. Prevalence of viruses were not similar overall (*χ*^2^ = 42.5, DF = 3, *P* < 0.001), with IAPV as the only virus below 100% prevalence. There was a difference in prevalence based on a virus by bee source interaction (*χ*^2^ = 11.6, DF = 3, *P* = 0.009), which was broken down by virus type (Table 1). There were more detections of IAPV in the HV bees than LV bees. There were no other significant terms for prevalence, including cage type.

**Table 1.** Prevalence of honey bee viruses per 50 ng of cDNA in pooled bees from contact vs. aerosol experiment cages based on their source (HV (*n* = 20) or LV bees (*n* = 39); maximum likelihood). Sides of cages were excluded from analyses based on potential sample degradation (≥ 23.5 Ct values for beta actin); for HV cages 4 of 24 sides were excluded and for LV cages 9 of 48 sides were excluded.

	Caged bee source						
Viruses	HV bees (%)	*n*	LV bees (%)	*n*	*χ* ^2^	DF	*P*
BQCV	100.0	20	100.0	39	0.00	1	0.99
IAPV	23.0	20	6.7	39	18.33	1	<0.001
DWV-A	100.0	20	100.0	39	0.00	1	0.98
DWV-B	100.0	20	100.0	39	0.00	1	0.98

To examine possible spread of viruses between HV donor and LV acceptor sides of cages, we looked at 2 parameters: difference in virus gene copies between sides of all cage types, and, the slope of the difference between the 2 sides in the contact and aerosol cages relative to the slope of the difference between the 2 sides in control cages.

Differences in virus levels were first examined in the 3 main effect treatments between sides of cages using mixed linear models including both weeks when trials were conducted. There was a significant interaction between treatment, side of cage, week, and virus in the fully specified model; thus, we analyzed each virus separately (*F*_6,87.1_ = 2.7, *P* = 0.020). There were significant interactions between treatment and side within viruses for BQCV (marginally; *F*_2,12_ = 3.6, *P* = 0.059), IAPV (*F*_2,9.81_ = 4.4, *P* = 0.044), and DWV-A (*F*_2,17.1_ = 5.9, *P* = 0.012), but not for DWV-B (*F*_2,20_ = 3.0, *P* = 0.071). Consequently, we performed contrasts to compare treatment changes relative to controls for only BQCV, IAPV, and DWV-A.

**Fig. 8.**
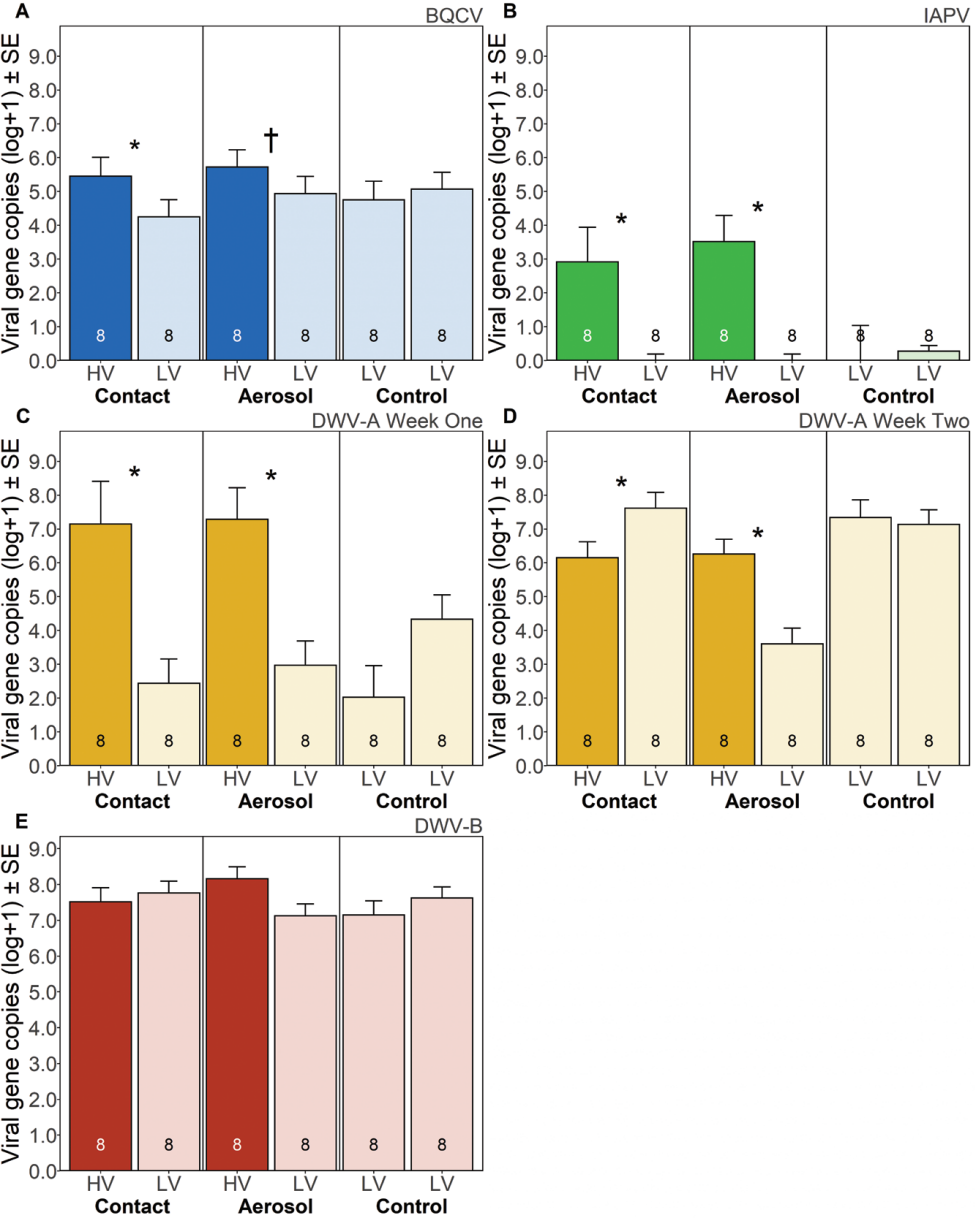
Potential effect of contact or aerosol transmission between groups of adult bees within cages after 6 d of exposure. Mean abundance ± SE of (A) BQCV (black queen cell virus), (B) IAPV (Israeli acute paralysis), (C) DWV-A (deformed wing virus, strain A) week one, (D) DWV-A week two, and (E) DWV-B (deformed wing virus, strain B) gene copies per 50 ng of cDNA from the contact vs. aerosol cage study (only including samples with ≤ 23.5 Ct for beta actin). Each bar shows the source of bees (HV for high virus bees in the darkest colors, LV for low virus bees in the lighter colors) and the 3 treatment cage types: aerosol (double screen), contact (single screen), and control (double screen with both sides containing LV bees; all bars *n* = 8). Plotted data are based on least squares means. An asterisk (*) or cross (†) denotes a significant or marginally significant difference (respectively) within sides of a treatment cage relative to the difference between sides in control cages (*P* < 0.05 and *P *< 0.06: repeated-measures ANOVA, Tukey HSD).

Levels of BQCV were higher in HV versus LV sides in contact cages (*F*_1,12.8_ = 7.4, *P* = 0.018: Fig. 8A). There was a marginally significant difference between sides for aerosol cages (*F*_1,11.5_ = 4.5, *P* = 0.056). There was no difference in BQCV between sides of control cages (*F*_1,11.7_ = 0.6, *P* = 0.44).

IAPV levels were also higher in HV sides of cages for both treatments (contact, *F*_1,10.9_ = 7.9, *P* = 0.017; aerosol, *F*_1,8.28_ = 19.7, *P* < 0.002: Fig. 8B), and there was no difference in sides of control cages (*F*_1,10.2_ = 0.1, *P* = 0.80).

For DWV-A, there was an interaction between week, treatment, and side (*F*_2,18_ = 15.9, *P* = 0.001), therefore, DWV-A was analyzed separately by week. Treatment by side interactions were present in both week one (*F*_2,3.35_ = 11.9, *P* < 0.030) and 2 (*F*_2,4.01_ = 31.6, *P* < 0.004). Levels of DWV-A in contact cages were higher in HV sides than LV sides in week 1 (*F*_1,4.08_ = 11.6, *P* = 0.026; Fig. 8C), however, HV sides were significantly lower than LV sides in week 2 (*F*_1,4.32_ = 12.6, *P* = 0.021; Fig. 8D). Levels of DWV-A in aerosol cages were higher on HV sides in both week 1 (*F*_1,3.03_ = 15.6, *P* = 0.028) and week 2 (*F*_1,3.73_ = 63.4, *P* = 0.002). There was no significant difference between sides of control cages in week 1 (*F*_1,3.03_ = 4.5, *P* = 0.12) or in week 2 (*F*_1,3.97_ = 10.7, *P* = 0.65).

As noted above, there was no interaction between treatment and side for DWV-B (*F*_2,20_ = 3.0, *P* = 0.071: Fig. 8E) or any other effect.

## Discussion

This study provides evidence of honey bee viral sequences on wax comb from winter loss colonies and the first evidence of bee-mediated introduction of viral sequences to wax through worker traffic. These findings indicate that wax could have an important and heretofore neglected function in honey bee virus epidemiology. Additionally, we present the first evidence of the aerosolization and airborne nature of honey bee viruses. Our evidence revealed that virus particles spread within an incubator and contaminated wax in the absence of direct contact with bees. This aerosolization did not affect the viral status of adult worker bees, suggesting that it may not be of concern as a factor in cage studies with adult bees. Nevertheless, the aerosolization of honey bee viruses, as well as the occurrence of waxborne honey bee viruses, merit further investigation as potential transmission to larvae via aerosolization and to bees from aerosol-contaminated wax have not been studied. Furthermore, there is potential for wax contamination by aerosol to occur in bee storage facilities which merits more study. We have provided evidence for the existence and introduction of BQCV through worker traffic on comb surfaces, the aerosolized deposition of virus particles onto comb, and the persistence of BQCV, IAPV, DWV-A, and DWV-B on comb in the absence of live bees.

### Winter Loss Wax Study

Our results showed that there were detectable and quantifiable levels of honey bee viruses on wax of winter loss colonies. We tested wax from colonies that died during indoor wintering between the months of November to April and showed that BQCV, IAPV, DWV generic, and the strains DWV-A and DWV-B were present when colonies were sampled in spring. We do not have exact times of death for colonies in this experiment, but know they failed sometime during winter and thus could have had no live bees present to introduce fresh virus onto wax weeks or months before testing. This suggests that waxborne viruses persist on wax and are detectable without constant re-introduction from worker traffic and other activities. Our samples of wax likely also included other material such as silk cocoons, which could have been a source of virus detection; nevertheless, our worker traffic experiment showed waxborne viruses in the absence of these materials.

Viruses differed significantly in both prevalence and abundance, with a general pattern of the DWV complex being detected at higher rates and levels, followed by BQCV and then IAPV. It could be that these differences reflect the levels in bees that initially deposited the viruses onto colony wax, as we found proportional transfer of BQCV in the worker traffic cage study. BQCV and DWV are nearly ubiquitous in Canadian colonies, while IAPV has lower prevalence (Desai et al. 2015); IAPV was also least prevalent in our contact vs. aerosol cage study. We suggest an additional factor could also be contributing to the differences in prevalence and abundance among BQCV, DWV complex, and IAPV: the rate of virus degradation specific to type. Other work has shown differences in the stability of viruses on wax over time and with irradiation. For example, though DWV, BQCV, and IAPV were all reduced by time and e-beam irradiation, IAPV was not detectable in any samples post-treatment even without irradiation, whereas BQCV and DWV were (Colwell 2022a). Further work should be done on the longevity and relative stability of viruses on wax comb in order to develop successful mitigation strategies against waxborne viruses for honey bees. It was also intriguing that the levels of DWV-A and DWV-B were not additively equivalent to levels detected by the generic DWV primer. It is possible that there was some overlap in detections by DWV in conserved areas of the strain genomes.

With the exception of IAPV, all waxborne viruses were positively correlated with each other. This may be due to virus infections within living colonies, which we believe is more plausible than interactions among the viruses on wax. A recent study of pathogen webs in Canadian colonies showed that BQCV, DWV-A, and DWV-B were positively correlated with each other in adult bees in fall and in the next spring (Borba et al. 2022). Single forager bees also exhibited a positive correlation between BQCV and DWV-A and B strains (D’Alvise et al. 2019). IAPV was not correlated with either BQCV or DWV in a further study that included indoor wintering (Desai and Currie 2016); however, the same study did not observe correlations between BQCV and DWV. It is also possible that the low detection rate and abundance of IAPV precluded any correlation from coming to light. In future work, it would be interesting to be able to compare levels of viruses in adult worker bees prior to colony death to levels of waxborne viruses detected post-colony death.

The presence of waxborne viruses on winter loss colony wax needs to be considered when assessing the potential impacts on colonies established on virus-infected comb and could be relevant for beekeeping practices, such as swapping frames among colonies to balance populations. If viruses are still detectable after time without continual re-introduction, it could be possible that persisting waxborne viruses can affect bees housed on contaminated comb following winter losses.

### Worker Traffic Cage Study

We found that BQCV was detected on wax foundation after experimental exposure to adult worker bees, providing evidence that contact with adult bees is sufficient to transmit viruses onto wax. We termed this exposure as “worker traffic” because it resulted exclusively from workers living and walking on wax in absence of in-hive activities such as brood care and food storage. Although we did not notice signs of defecation or regurgitation of food in our cage studies we cannot fully discount that this could have contributed to the deposition of virus on comb. We only tested for BQCV during this experiment, but other viruses are likely introduced in a similar fashion (ie IAPV and DWV complex detected on winter loss wax as above).

There is existing evidence of honey bees introducing viruses to their environments. BQCV and DWV have been discovered on pollen pellets carried by foraging bees, and furthermore, DWV in bee bread was infective when fed to naïve colonies (Singh et al. 2010). Similarly, DWV was found in the pollen of honey bee-visited flowers, and the pollen proved to be infective to bees via injection (Mazzei et al. 2014). BQCV and DWV-infected colonies were found to deposit both those viruses on flowers while foraging, and were more likely to deposit viruses during longer and more frequent visits (Alger et al. 2019). There may be multiple routes of infection from contaminated wax, including contamination of stored food or potentially by direct contact with wax by brood or adult bees.

In addition to being found on wax, BQCV was universally prevalent in bees from each treatment. There was a strong correlation between the viral levels of bees and the amount of virus found on associated wax, seen in the relationship between pooled HV and LV bees and their associated wax. A study examining the effect of gamma irradiation on wax had similar results, with DWV-B (termed as VDV-1) levels on wax highly correlated with levels in bees (de Guzman et al. 2019). These findings provide strong evidence not only that honey bee viruses can be introduced to wax by worker traffic, but that the introduction of viruses is proportional to the level of viruses in those bees. We did not correlate BQCV levels in bees with varroa infestation levels, as bees tested for BQCV were already homogenized within the HV and LV treatments for cages. Though there were higher BQCV levels in the HV versus LV treatment, this does not necessarily contradict findings that BQCV is not correlated with varroa (Emsen et al. 2015), as our HV bee source bees could have had elevated BQCV for other reasons than high varroa levels.

Similar to Schittny et al. (2020) who found that DWV-A-contaminated hive products, including wax, can act as a route of virus transmission, we have shown there is potential for other waxborne viruses to infect bees from worker-contaminated comb. The results of our study suggest that more highly infected bees (ie those with higher virus levels) contaminated wax with BQCV to a greater extent. Therefore, colonies containing bees with high virus loads may be more likely to contaminate colony wax, though more work is needed to determine if this could affect colony health. The bees sampled from source colonies for our cage experiments were not newly emerged, and therefore could have obtained viruses from different sources within their colonies. However, as all bees came from a situation of possible pre-exposure to viruses and we compared relative differences between groups of pooled bees, we are confident that this would not have impacted our results.

We were surprised to discover that BQCV was present on wax samples from cages that did not contain any bees. These were exposed to the same temperature and humidity conditions inside the incubator as cages containing bees, and any potential degradation of virus would also be similar. There was also a significant difference between levels of BQCV contained in the controls within, and exterior to, the incubator. Although, wax foundation outside the incubator was at a lower temperature (room temperature) and likely exposed to lower humidity, storage experiments (Colwell et al. 2024) suggest this would not significantly affect degradation in the time frame of the experiment. We believe this is the first evidence to suggest that BQCV can be aerosolized by worker bees within an incubator. This is very interesting, considering most conventional wisdom on horizontal transmission of honey bee viruses focuses on direct contact from bee to varroa or from bee to bee. Nevertheless, a recent study detected spores of *Nosema* spp., gut-infecting microsporidians, within air samples using spore-trapping tape (Sulborska et al. 2019). Additionally, a virus (a bacteriophage of *Escherichia coli* (Migula)) was found to be adsorbed by bees during flight in a wind tunnel (Lighthart et al. 2005). Previous studies and reviews have mentioned the possibility of airborne virus transmission within incubators but without conclusive answers (Bailey et al. 1980, Chen and Siede 2007, Amiri et al. 2014, 2019). The aerosolization of honey bee viruses presents a new avenue for studying honey bee virus transmission that should be further investigated. Though we provide evidence, discussed below, that aerosolized viruses do not affect adult bees under incubator conditions, we cannot yet rule out the possibility of aerosolized viruses affecting other aspects of colony life (ie possible effects on different life stages of bees, contamination of food stores, etc.). It is possible that positive detections from wax do not entirely represent viable viruses, rather that they could be detecting genetic material from partially intact viruses that could be detected by our primers. Nevertheless, waxborne viruses introduced by worker bees can affect virus levels in bee brood reared upon that wax; adults reared on contaminated wax had higher virus levels compared to those reared on wax with no detectable waxborne viruses (Colwell 2022b). However, further testing is needed to determine if wax contaminated by aerosolized viruses has an effect on honey bee health. It is theoretically possible that airborne viruses could have effects on horizontal transmission among colonies in the context of mass storage and transport of colonies over long periods of time, such as in overwintering scenarios and migratory beekeeping. Future work should investigate longer term exposures and the potential of other viruses (eg DWV, IAPV) to become aerosolized.

### Contact vs. Aerosol Cage Study

In a logical extension of evidence that BQCV aerosolized within an incubator in the above worker traffic cage study, we further tested the implications for worker to worker transmission of viruses through contact or aerosolization. BQCV and DWV strains were present in all pooled samples, while IAPV was detected at lower prevalence, similar to our winter loss wax analysis and published data showing more variability in IAPV than BQCV or DWV in the study region.

We tested the potential of high varroa (HV) bees to affect the virus load of low varroa (LV) bees via shared air, incorporating treatments with potential for aerosol transmission, contact transmission, and a control. We hypothesized that, if aerosol transmission was seen, virus levels on LV sides of aerosol cages would be higher relative to control cages. However, in contrast to our findings which showed evidence of deposition of BQCV on wax through aerosol transmission, there was no clear evidence that aerosol transmission from HV to LV bees affected the viral level of LV bees for the tested viruses (BQCV, IAPV, DWV-A, and DWV-B) within the 6-d time frame of the experiment. It is important to note that the mesh separating the 2 sides of the cages was fine enough to prevent varroa from crossing through.

For BQCV, we did observe fairly consistent differences between HV and LV sides in aerosol cages when compared with controls indicating our virus level treatment manipulation was largely successful. However, if there was convincing evidence of virus spread from worker to worker by aerosol transmission, we would have expected: (i) LV sides to not differ from the HV sides, (ii) the relative difference between HV and LV to be similar to the contact treatment, and (iii) the LV sides in the aerosol treatment to be greater than the LV controls. As such, we did not find evidence of aerosol transfer. Although BQCV did not differ between sides in aerosol cages, it did differ in contact cages, which had the same sharing of air. Coupled with the evidence that the difference between sides in aerosol cages was marginal, we do not believe there is biological significance to this finding.

DWV-A was at significantly higher levels in the second week, which we suggest could be due to the seasonal upturn of viruses leading into autumn. A French study showed DWV prevalence was significantly higher in autumn compared to spring and summer in both adults and pupae (Tentcheva et al. 2004). However, their study did not differentiate by DWV strain, and we did not see an effect of week (or any main effect) in DWV-B levels in our samples. Levels of DWV-A were also unexpectedly higher on LV sides of contact cages in week 2, in line with higher levels in controls cages; conversely, LV sides in aerosol cages were lower than those controls. It could be that there was competition of viruses within HV bees and the high levels of other viruses and strains inhibited DWV-A from replicating.

We believe the duration each trial over 6 d was reasonably sufficient to show cross cage infections. There were various considerations for keeping the experiment to this duration. The potential for contamination of cages was high when the bags were opened, and doing so was limited to replacing food and water. The build up of dead bees through the course of the experiment and the impending start of autumn also influenced restricting each experimental trial to a length of 6 d. Mortality was not measured day-to-day to avoid potential contamination and limit disconnection from the air source and was not anticipated to be significant given the short duration of the cage trials. Mode of transmission is an important factor in the speed with which viral infections become established, for example, DWV artificially injected into adult bees caused overt infections, compared with covert infections resulting from orally fed DWV over 3 d (Mockel et al. 2011). Caged adult bees fed a mixed inoculum of viruses did, however, show infections of BQCV, DWV, and IAPV at 12 and 36 h post feeding (Carrillo-Tripp et al. 2016). Other work focusing on individual viruses detected physical symptoms of IAPV (Amiri et al. 2019), and molecular detection of DWV (Mockel et al. 2011, Thaduri et al. 2019), only 3 d after inoculation or half the time of our cage experiments. However, viruses injected by a vector can develop faster than when transmitted through indirect means. For example, BQCV fed to adult bees resulted in lower levels at 10 d compared to the same dose of BQCV after injection, mimicking parasite vectoring, into adult bees (Al Naggar and Paxton 2020). It may be possible that aerosolization of viruses is a horizontal transmission route that takes even longer to present as an overt infection. As such, additional experiments over longer time frames are needed to address this possibility.

We cannot discount the idea that air flow from the air compressor into each bag interfered with the flow between the sides of cages. We detected aerosolized spread onto wax in the worker traffic cage study when there was no extra input of air into the incubator. Air flow into the cage bags may have prevented a natural exchange of air between the 2 sides, precluding our intended results; however, it was the best method available at the time of the experiment to prevent contamination among cages within the incubator. To better examine cross-cage transmission by contact, future experiments could force interaction by providing only one side with food to boost trophallaxis within cages (Free and Butler 1958). A cage experiment that mixed Acute bee paralysis virus (ABPV)-injected bees with naïve bees did not cause transmission to the naïve bees even when they fed from the same food source; however, ABPV was transmitted when the injected bees engaged in trophallaxis with the naïve bees (Bailey and Gibbs 1964). Further work could also examine aerosol and contact transmission of a virus present only on one side of cages to completely naïve bees, including cages with wax only on one side to test aerosol deposition in conjunction with bee-bee transmission.

Nevertheless, more experiments should be done to determine if aerosolized honey bee viruses have an effect on honey bee health at different life stages. Although we found evidence suggesting BQCV may aerosolize within an incubator in our worker traffic study, this experiment shows aerosolized viruses are likely not an important confounding factor when conducting viral studies on caged adult workers in incubators. These results also raise concerns about common beekeeping practices, particularly the reuse of potentially contaminated equipment following removal from winter storage or interchanging frames among colonies at any time. Overall, our findings increase our understanding of the epidemiology of honey bee virus transmission within the hive environment. Clearly, more attention is needed to the less obvious routes of honey bee virus transmission.

## Supplementary Material

ieaf037_suppl_Supplementary_Figures_S1-S5

ieaf037_suppl_Supplementary_Tables_S1

ieaf037_suppl_Supplementary_Tables_S2

ieaf037_suppl_Supplementary_Tables_S3
