# Supplementary material for: Mechanical transfer of honey bee (Hymenoptera: Apidae) virus sequences to wax by worker traffic and aerosolization
Source: J Insect Sci. 2025 May 22;25(3):9. doi: 10.1093/jisesa/ieaf037 (PMC12096080; doi:10.1093/jisesa/ieaf037)
Supplement: ieaf037_suppl_Supplementary_Figures_S1-S5 [file ieaf037_suppl_supplementary_figures_s1-s5.docx]

**BQCV**

**A.**

**
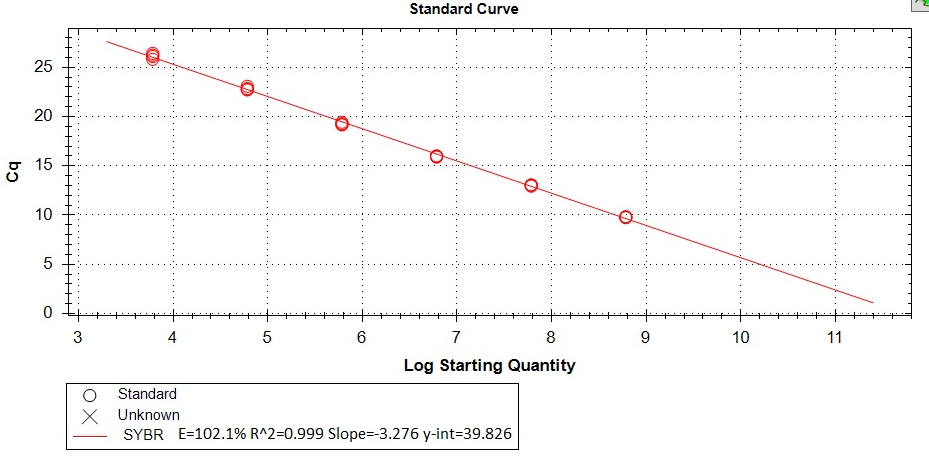
**

**B.**

**C.**

**
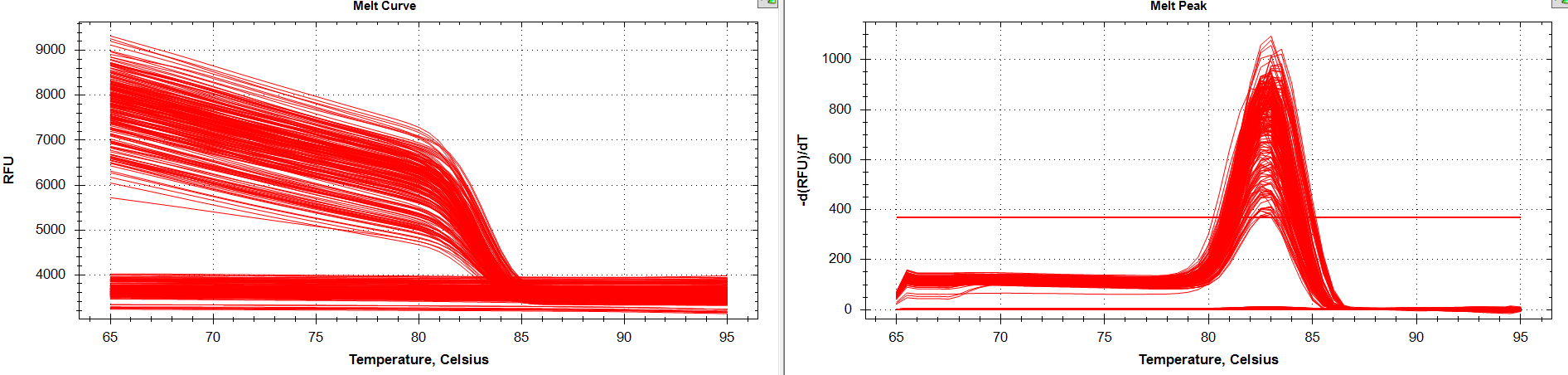
**

Figure S1. Example RT-qPCR readouts for BQCV. A) standard curve for calculating starting virus quantity, B) melt curve for sample wells, C) melt peaks for sample wells.

**IAPV**

**A.**

**
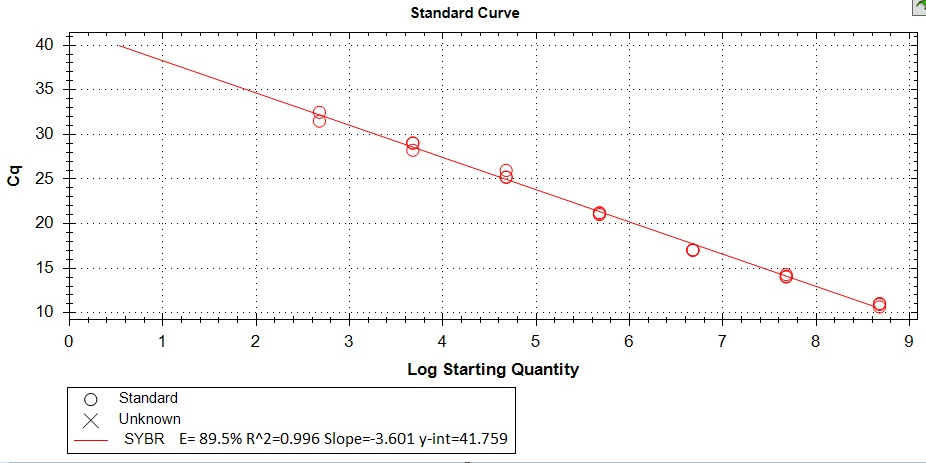
**

**
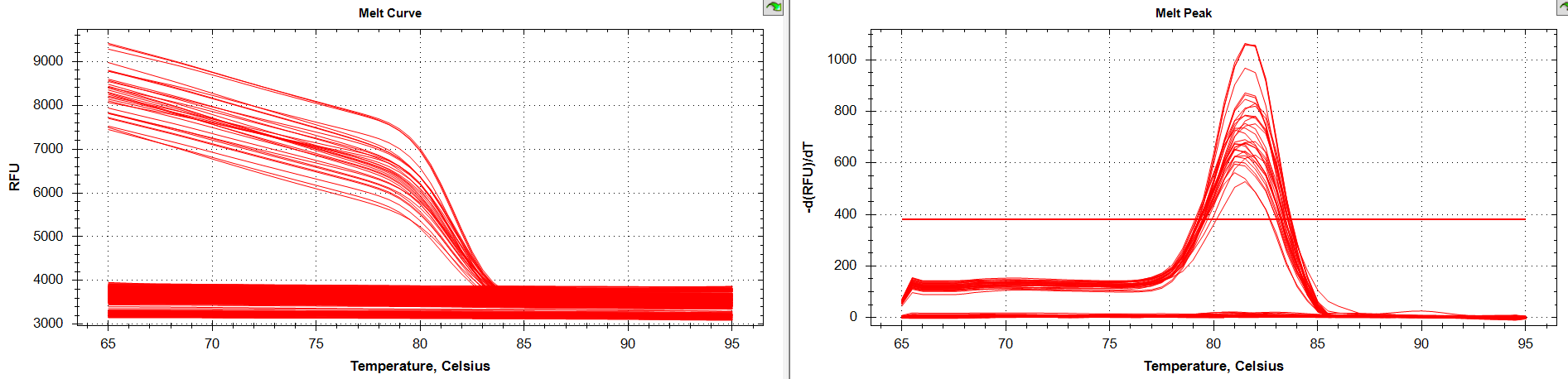
**

**C.**

**B.**

Figure S2. Example RT-qPCR readouts for IAPV. A) standard curve for calculating starting virus quantity, B) melt curve for sample wells, C) melt peaks for sample wells.

**DWV generic**

**A.**

**B.**

**C.**

**
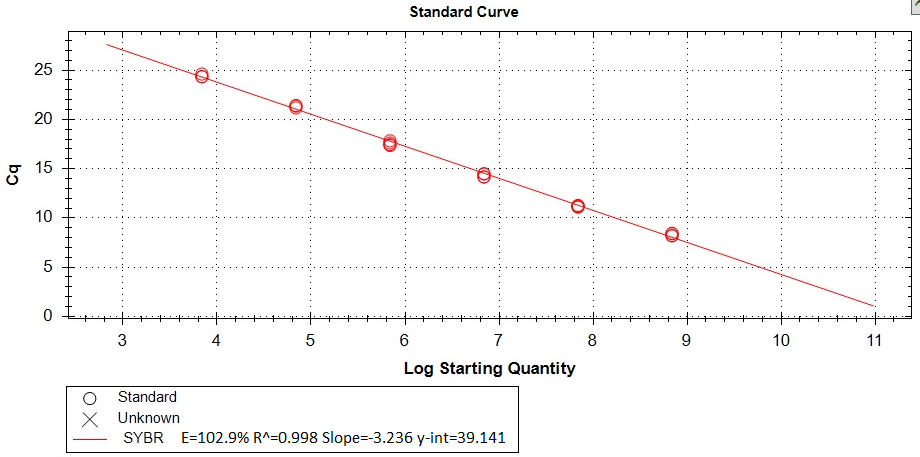
**

**
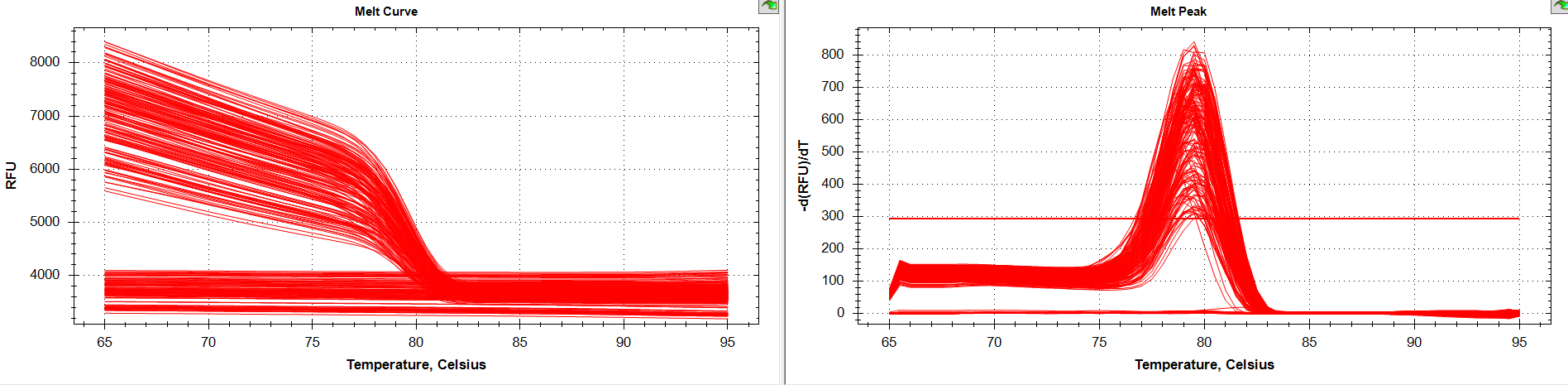
**

Figure S3. Example RT-qPCR readouts for DWV generic. A) standard curve for calculating starting virus quantity, B) melt curve for sample wells, C) melt peaks for sample wells.

**DWV-A**

**A.**

**
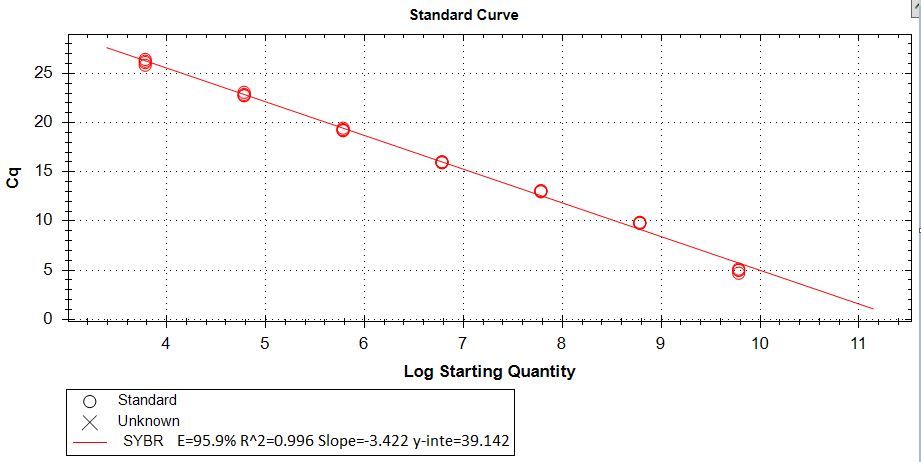
**

**C.**

**B.**

**
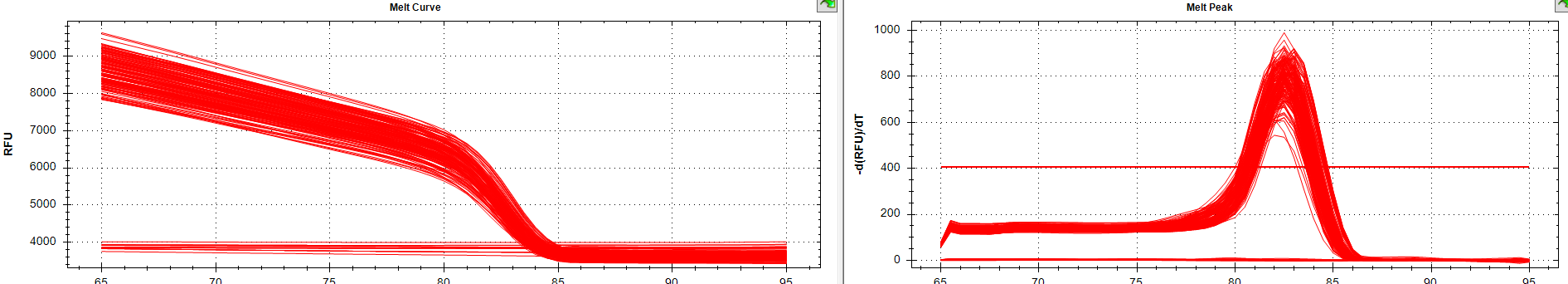
**

Figure S4. Example RT-qPCR readouts for DWV-A. A) standard curve for calculating starting virus quantity, B) melt curve for sample wells, C) melt peaks for sample wells.

**DWV-B**

**A.**

**
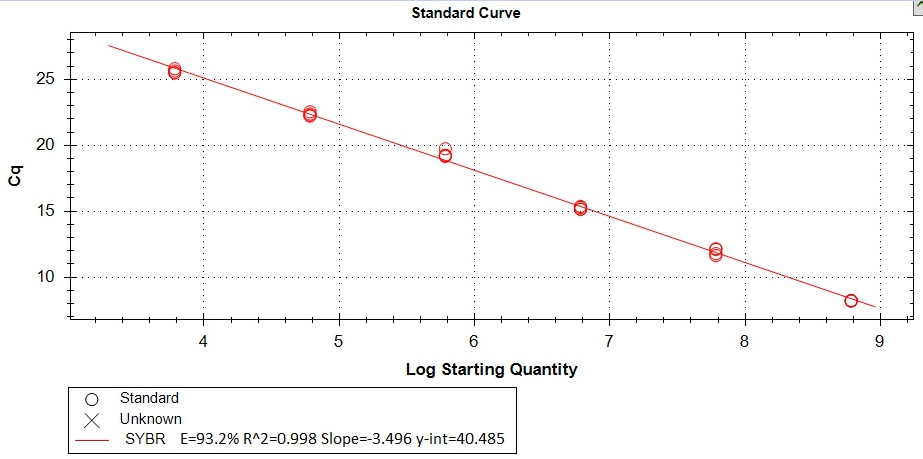
**

**C.**

**B.**

**
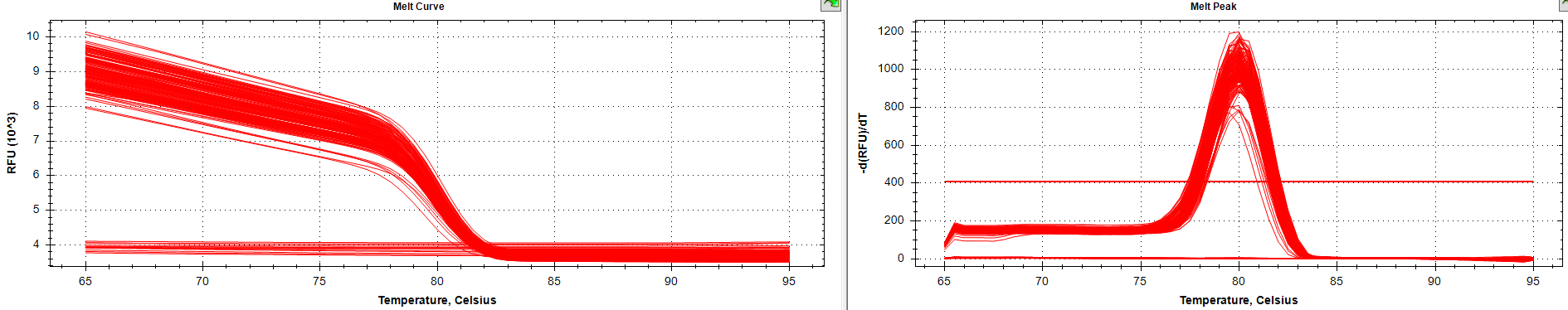
**

Figure S5. Example RT-qPCR readouts for DWV-B. A) standard curve for calculating starting virus quantity, B) melt curve for sample wells, C) melt peaks for sample wells.
