# Supplementary material for: Mechanical transfer of honey bee (Hymenoptera: Apidae) virus sequences to wax by worker traffic and aerosolization
Source: J Insect Sci. 2025 May 22;25(3):9. doi: 10.1093/jisesa/ieaf037 (PMC12096080; doi:10.1093/jisesa/ieaf037)
Supplement: ieaf037_suppl_Supplementary_Tables_S1 [file ieaf037_suppl_supplementary_tables_s1.docx]

| Target | Forward Primer | Reverse Primer | Product size (bp) | Source |
| --- | --- | --- | --- | --- |
| β-Actin-181 | AGGAATGGAAGCTTGCGGTA | AATTTTCATGGTGGATGGTGC | 181 | 2 |
| BQCV-310 | CCTGTATTCATGCATCTCAGA | GCAACAAGAAGAAACGTAAACCAC | 310 | 1 |
| IAPV-586 | GCGGAGAATATAAGGCTCAG | CTTGCAAGATAAGAAAGGGGG | 586 | 3 |
| DWV/VDV-1 221 | GGGTGCGTAAATATGGTGG | TAGTATCTGAAACAGCTTCC | 111 | 4 |
| DWV-A | TTCATTAAAGCCACCTGGAACATC | TTTCCTCATTAACTGTGTCGTTGA | 136 | 5 |
| DWV-B | TATCTTCATTAAAACCGCCAGGCT | CTTCCTCATTAACTGAGTTGTTGTC | 139 | 5 |

Table S1. Primer sequences used in the present experiments for the housekeeping gene (β-Actin) and quantification of virus types by RT-qPCR.

**REFERENCES**

1. Benjeddou, M., Leat, N., Allsopp, M. and Davison, S., 2001. Detection of acute bee paralysis virus and black queen cell virus from honeybees by reverse transcriptase PCR. Appl Environ Microbiol 67:5, 2384-2387. 10.1128/AEM.67.5.2384-2387.2001.
2. Chen, Y. P., Higgins, J. A. and Feldlaufer, M. F., 2005b. Quantitative real-time reverse transcription-PCR analysis of deformed wing virus infection in the honeybee (Apis mellifera L.). Appl Environ Microbiol 71:1, 436-441. 10.1128/AEM.71.1.436-441.2005.
3. Di Prisco, G., Pennacchio, F., Caprio, E., Boncristiani, H. F., Jr., Evans, J. D. and Chen, Y., 2011. Varroa destructor is an effective vector of Israeli acute paralysis virus in the honeybee, Apis mellifera. J Gen Virol 92:Pt 1, 151-155. 10.1099/vir.0.023853-0.
4. Ryabov, E. V., Wood, G. R., Fannon, J. M., Moore, J. D., Bull, J. C., Chandler, D., Mead, A., Burroughs, N. and Evans, D. J., 2014. A virulent strain of deformed wing virus (DWV) of honeybees (Apis mellifera) prevails after Varroa destructor mediated, or in vitro, transmission. PLOS Pathog 10:6, e1004230. 10.1371/journal.ppat.1004230.
5. Traynor, K. S., Rennich, K., Forsgren, E., Rose, R., Pettis, J., Kunkel, G., Madella, S., Evans, J., Lopez, D. and vanEngelsdorp, D., 2016. Multiyear survey targeting disease incidence in US honey bees. Apidologie 47:3, 325-347. 10.1007/s13592-016-0431-0.
