# Supplementary material for: Mechanical transfer of honey bee (Hymenoptera: Apidae) virus sequences to wax by worker traffic and aerosolization
Source: J Insect Sci. 2025 May 22;25(3):9. doi: 10.1093/jisesa/ieaf037 (PMC12096080; doi:10.1093/jisesa/ieaf037)
Supplement: ieaf037_suppl_Supplementary_Tables_S2 [file ieaf037_suppl_supplementary_tables_s2.docx]

| Target | gBlock sequence |
| --- | --- |
| BQCV-310 | GAATTCGGAAACATTTTACTATAGTTCAGGTCGGAATAATCTCGATATAGCCACTTCACCTCCCACCGTCAATCGCTATTATGCGGTTGGTGCGGGAGATGATATGGACTTTTCCATCTTTATCGGTACGCCGC**CCTGTATTCATGCATCTCAGA**CGGCCCAGTTTACCAAGATAAAACAAGGTAAAGTGTATGATTTGAGGTATGATCAGTACGACCCTTTTAGGGAAGTCCAGGACGGTACGGCGTTCCTCAATGCTCGTAGTATTGAGGATAGCGATTTGTTGTGAGCTCCTTTAGAGGGAGGGCTCACTTTATCTATTGCTTAATTCGGTAAGCCACAAATTTTTCTAAGTGTCATGAGTTTCTTCTCGGTTCTTCTCATGATTACTAATCGAACCGTGTGTAGAGTCAGAATGTT**GTGGTTTACGTTTCTTCTTGTTGC**TTCGAGTTAAAGTAGTTACTGAGAAGGGTGTAGATTTCGTCAGAGC |
| IAPV-586 | GATTACACCCCCGTATGGA**GCGGAGAATATAAGGCTCAG**CTAGGATGACACGCCTGTATACTGTCGATATTAGTTAAGTTGCAATTACACGTCTGTTGCCGAAGAAACCATTTTAGTCAACTGATTATGATTTTTGTATAATGACAAACAGTGATGAACTGTATAACTCATCAATAACAAAACGGATTACGAACCTATTTGTAACTATCTTGATCGAAGTCCAGTAGATCCCCAATATAGCCCTGAAAAGCTTGAGGGACGAGATAGCTCTATAGATAGACGTGAGGCTTTAAATCCTGATAAGTACATTACCTGAGAATTCCTCCTTTTGGAGTTTGAATTTATATAAGTTAGTACCAATAGTTAATATCATTTAAGTATGTTATTATTGCTGAAGGCATGTATTTCGGATAATAACCTCTACATTGATATATAAACTATATGCAAGTCTCGGTGGATATTGCGTTATGGTCGCAGTTAACCTGTAGCTTATATATTCCTGTGTCGGAGCAGTGGTAATGGAGCCGGACAATTTCGCCAAAAATGTTTACTTCTCAACAAAACAATAATATCACCAAACAACCCGC**CCCCCTTTCTTATCTTGCAAG**TATGCGAGCAAGATTACA |
| DWV/VDV-1 221 | GATTACAGTGCCCGGTTTGACAGCATCTTTGCAACTTCAAATGGACTATATGAAATTGAAGTCATCGAGTTATGTAGTATTTGATTTACAAGAAAGTAATAGCTTCACTTTTGAGGTGCCATAYGTTTCATATAGACCATGGT**GGGTGCGTAAATATGGTGG**YAATTATTTACCCTCGTCAACTGACGCTCCTAGTACATTATTTATGTATGTKCAGGTWCCRTTGATACCTAT**GGAAGCTGTTTCAGATACTA**TTGATATCAATGTGTACGTACGGGGCGGTAGTTCATTTGAAGTTTGTGTTCCAGTCCAACCTAGTTTAGGTTTGAATTGGAATACAGACTTTATTTTACGTAATGAYGAAGAATACAGGGCTAAGACAGGTTATGCACCATATTATGCTGGAGTGTGGCATAGCTTCAATAATAGTAATGATTACA |
| DWV-A | GTAGTTAAACCAATAAATGGTTGCAAGATTAGAAGTTTGCAAGATGCTATATGTGGTGTGCCTGGTTTAGATGGGTTTGATTCGATATCTTGGAATACTAGTGCTGGTTTTCCTTTGTC**TTCATTAAAGCCACCTGGAACATC**AGGCAAGCGATGGTTGTTTGACATTGAGCTACAAGACTCGGGATGTTATCTCCTGCGTGGAATGCGTCCCGAACTTGAGATTCAATTA**TCAACGACACAGTTAATGAGGAAA**AAGGGAATAAAACCTCACACTATATTCACGGATTGTTTGAAAGATACTTGTTTGCCTGTTGAAAAATGTAGAATACCTGGTAAGACTAGAATATTTAGTATAAGTCCGGTACAGTTTACCATACCGTTTCGACAGTATTACTTAGACTTTATGGCATCCTATCGAGCTGCACGACTTAATGCTGAGCATGGTATTGGTATTGATGTTAACAGCTTAGAGTGGACAAATTTGGCAA |
| DWV-B | TCCGTTGAAGTTAGGGTGTGAGAAACATGGTATGCCATGTTCTCCATTTAATCGAAAACATTTGGAATTAGCAACGACTCATTTAAAGGAGAAGTTAATTTCCGTAGTTAAACCTATAAACGGATGCAAGATTAGAAGTTTGCAAGATGCTGTGTGTGGTGTACCAGGTTTGGATGGCTTTGATTCAATATCCTGGAATACTAGTGCTGGTTTTCCTT**TATCTTCATTAAAACCGCCAGGCT**CTTCTGGTAAGCGATGGTTGTTTGATATTGAATTACAAGATTCAGGATGTTATCTTTTGAGAGGGATGAGACCTGAACTTGAGATACAGTT**GACAACAACTCAGTTAATGAGGAAG**AAGGGAATGAAGCCTCACACTATATTCACGGATTGTTTGAAAGATACATGTTTGCCTGTGGAAAAATGCAGAATACCTGGTAAGACTAGAATATTTAGTATAAGTCCCGTCCAATTTACGATTCCATTCCGA |

Table S2. gBlock sequences used to create standard curves for the quantification of virus types by RT-qPCR. Primer pairs are bolded and underlined.
