# Supplementary material for: Mechanical transfer of honey bee (Hymenoptera: Apidae) virus sequences to wax by worker traffic and aerosolization
Source: J Insect Sci. 2025 May 22;25(3):9. doi: 10.1093/jisesa/ieaf037 (PMC12096080; doi:10.1093/jisesa/ieaf037)
Supplement: ieaf037_suppl_Supplementary_Tables_S3 [file ieaf037_suppl_supplementary_tables_s3.docx]

| Target | Product sequence from this study | Identity (%) | Accession # | Position in genome |
| --- | --- | --- | --- | --- |
| BQCV | GGCGGCCCAGTTTACCAAAATAAAACAAGGTAAAGTGTATGATTTGAGGTATGATCAGTATGACCCTTTCAGGGAAGTCCAGGACGGTACGGCGTTCCTCAATGCTCGTAGTATTGAGGATAGCGATTTGTTGTGAGCTCCTTTAGAGGGAGGGCTCACTTTATCTATTGCTTAAATCGGTAAGCCACAAATTTTTCTAAGTGTCATGAGTTTCTTCTCGGTTCTTCTCATGATTACTAATCGAACCGTGTGTAGAGTCAGAATGTTGTGGTTTACGTTTCTTCTTGTTGC | 100.00 | KY243932.1 | 8223-8511 |
| IAPV | GTCGATATTAGTTAAGTTGCAATTACACGTCTGTTGCCGAAGAAACCATTTTAGTCAACTGATTATGATTTTTGTATAACGATAAACAGTGATGAACTGTATAACTCATCAATAATAAAATGGATTACGAACCTATTTGTAACTATCTTGATCGAAGTCTAGTAGATCCCCAATATAGCCCTGAAAAGCTTGAGGGACGAGATAGCTCTATAAATAGACGTGAGGCTTTAAATCCTGATAAGTACATTACCTGAGAATTCCTCCTCTTGGAGTTTGAATTTATATAAGTTAGTACCAATAGTTAATATCATTTAAGTATGTTATTATCGCTGAAGGCATGTATTTCGGATAATAACCTCTACATTGATATATAAACTATATGCAAGTCTCGGTGGATATTGCGTTATGGTCGCAGTTAACCTGTAGCTTATATATTCCTGTGTCGGAGCAGTGGTAATGGAGCCGGACGATTTCGCCAAAAATGTTCACTTCTCAACAAAATAATAAAATCACCAAACAACCCGCCCCCCTTTCTTATCTTGCAAG | 98.35 | EU224279.1 | 144-689 |
| DWV/VDV-1 221 | ACATAAATAANTGTANTAAGGACGGCGTCAGTTAGACGGATGGTAAATAATTGCCACCATATTTACGCACCCAAA | 89.04 | MT096529.1 | 3158-3224 |
| DWV-A | TTTCATTAAAGCCACCTGGAACATCAGGTAAGCGATGGTTGTTTGACATTGAGCTACAAGACTCGGGATGTTATCTTCTGCGTGGAATGCGTCCCGAACT | 98.99 | OM648742.1 | 8366-8464 |
| DWV-B | CGGTCTCNTCCCTCTCNAAGANAACATCCTGAATCCTGTAATTCAATATCAAACAACCATCGCTTACCAGAAGAGCCTGGCGGTTTTAATGAA | 94.62 | MN538209.1 | 8623-8715 |

Table S3. Sequences of amplified virus product for sequence identification by comparison to known viral genomes in NCBI.
